# Monoterpenoid-Based Deep Eutectic Solvents/Natural Deep Eutectic Solvents: A Review on Its Characteristics and Applications

**DOI:** 10.1021/acsomega.5c10885

**Published:** 2026-01-23

**Authors:** Felipe Gois Mota, Evertan Antonio Rebelatto, Laís Benvenutti, Elena Ibáñez, Sandra Regina Salvador Ferreira, Marcelo Lanza

**Affiliations:** † Department of Chemical and Food Engineering, Technological Center, 28117 Federal University of Santa Catarina, 88040900 Florianópolis, Brazil; ‡ Foodomics Laboratory, Instituto de Investigación en Ciencias de la Alimentación (CIAL, CSIC-UAM), Nicolás Cabrera 9, Campus de Cantoblanco, 28049 Madrid, Spain

## Abstract

The use of deep eutectic solvents (DES) and natural deep eutectic solvents (NADES) as green extraction solvents holds significant potential for various industrial applications. This review aims to summarize recent studies (2020–2025) on the use of sustainable solvents such as DES and NADES based on monoterpenoids in extraction processes. It also covers their production and other applications found in the literature such as extraction of natural compounds from raw materials, biorefinery, and the combination with supercritical carbon dioxide. According to the literature data, the use of green solvents can be improved through its combination with green extraction methods (ohmic heating, ultrasound, microwave, and supercritical technology). DES and NADES can be prepared in specific molar proportions with the aid of energy sources (conventional heating, microwave reactor, and ultrasound). DES/NADES composed of monoterpenoids show significant potential for industrial applications, particularly in the extraction of bioactive compounds, antioxidants, and natural pigments from food matrices. These solvents also hold potential for innovation and application across various sectors, including health, pharmacy, chemistry, environment, petrochemicals, biorefinery, metallurgy, and supercritical technology. Unexplored areas, such as the combination of monoterpenoid-based DES/NADES with supercritical carbon dioxide, the study of their phase equilibrium, and investigation of their thermophysical properties under varying temperature and pressure conditions, present interesting opportunities from a technological point of view.

## Graphical abstract

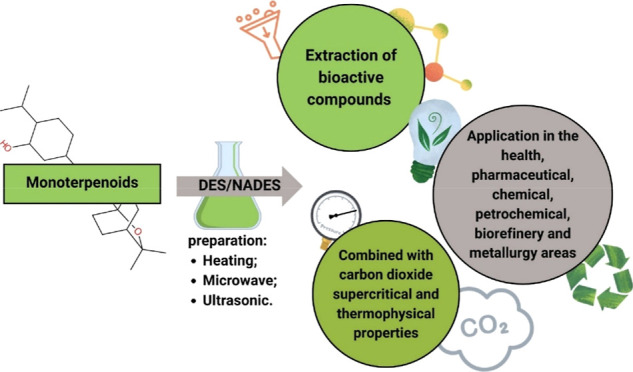

## 1 Introduction

Deep eutectic solvents (DES) are rapidly gaining attention as a sustainable alternative to traditional solvents. This increase in utilization can be attributed to their distinct properties, such as the ease of preparation and cost-effectiveness.^1,2^ Replacing hazardous solvents with emerging green solvents, such as DES, enables environmentally friendly processes for several sectors.^3,4^ These alternative solvents consist of a hydrogen bond acceptor (HBA) and one or more hydrogen bond donors (HBDs), forming a complex in which the electric charge is stabilized. Strong molecular interaction between the HBA and HBD components leads to a mixture with melting point lower than that of the individual components, exhibiting unique thermodynamic properties.^5,6^ By definition, a deep eutectic solvent (DES) must have a specific molar ratio of HBA and HBD that produces a eutectic temperature lower than the ideal eutectic temperature of this mixture.^7^

In general, DESs are considered biodegradable green solvents and exhibit low cytotoxicity; however, these properties are highly dependent on the structures of the HBA and HBD components.^2,8^ The use of organic acids as HBDs can negatively affect the biodegradability and increase the corrosivity of DESs; nonetheless, further investigation is needed to fully understand these effects.^8^ Natural deep eutectic solvents (NADES) are primarily composed of secondary metabolites or other compounds found in cells, which confer biocompatibility, making them ideal for the extraction of organic compounds.^9^ However, caution is warranted when using the term “natural” as compounds classified as natural, such as choline chloride and urea, are often produced synthetically on a large scale.^8^

Monoterpenes and monoterpenoids are phytochemicals utilized in the production of DES and NADES. The alternative solvents created from these hydrocarbons are progressively superseding traditional solvents for the extraction of bioactive substances from various raw materials.^10^ Most monoterpenoid-based NADES are low-viscosity, nontoxic, biodegradable, and cost-effective fluids that can be used to develop sustainable solvents for various processes and applications in various fields.^11^

Considering that several components and molar ratios can be combined to prepare DES and NADES, resulting in solvents with diverse properties, tools based on statistical mechanics, molecular dynamic, and quantum chemistry have been employed to correlate molecular structures with desirable solvent properties such as viscosity, solubility, toxicity, and melting point.^12,13^ Among these tools, conductor-like screening model for real solvents is based on quantum chemical calculations integrated with thermodynamic approaches and can be used to predict key parameters such as the chemical potential and solubility. Thus, these tools are essential for customizing DES and NADES for specific applications.^12,13^ Additionally, perturbed chain-statistical associating fluid theory (PC-SAFT) has been applied to DES and NADES to estimate the eutectic point of different component systems, considering hard-chain models and molecular associations.^14^ The use of DES and NADES as extraction solvents represents a potential strategy in several industries including nutraceuticals, pharmaceuticals, foods, and cosmetics.^15^

Therefore, the present review brings an evaluation on the recent studies (2020–2025) related to the use of green solvents such as DES and NADES, particularly those derived from terpenes and monoterpenes, presenting their preparation methods and application in extraction and other industrial processes. To address this subject, this review presents definitions for green solvents and green extraction as well as their preparation methods. Practical examples are presented to illustrate the potential of these solvents across various industrial sectors.

## 2 Green Solvents and Their Applications for Extraction Processes

The principles of Green Chemistry promote the development of eco-friendly practices, aiming to achieve a more sustainable society with less environmental impact.^16^ These principles contribute to improving clean energy production and consumption in the field of chemistry, promoting sustainable community development and industrial innovation.^17^ The use of green solvents and processes minimizes the environmental side effects associated with traditional methods, mostly because the alternative methods enable the reduction in the use of reagents, energy, equipment, and waste generation, which are key factors for procedure selection.^18^

When combined with green extraction technology, the selection of appropriate eco-friendly solvents becomes crucial since considerable amounts of solvents are typically used during the extraction processes.^19^ Notably, an exponential increase in the number of studies related to green solvents has been observed since 2004, with the highest number of publications recorded in 2024 (Figure 1).

**Figure 1:**
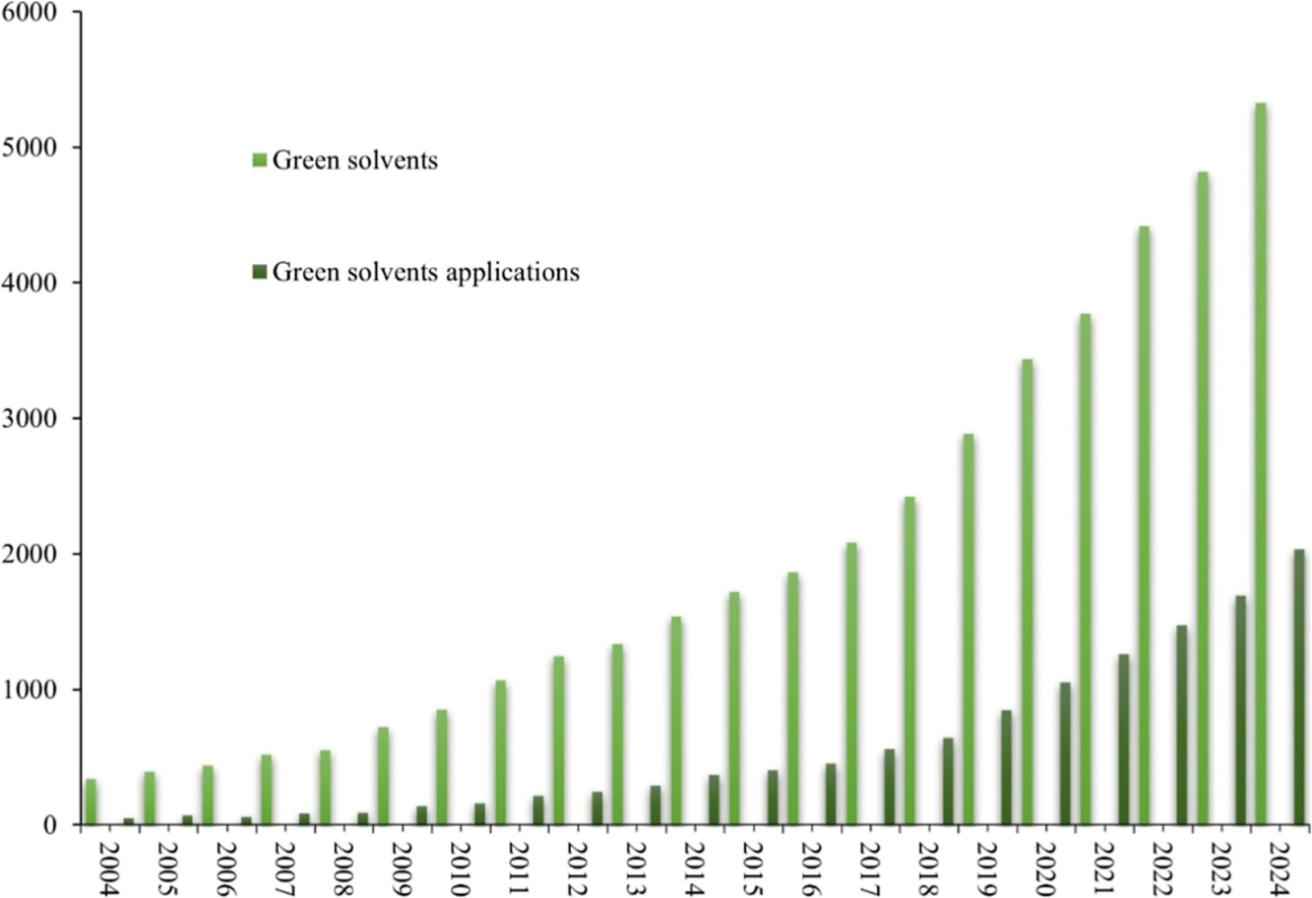
Number of studies on “green solvents” and “green solvent applications” published between 2004 and 2024 according to the SCOPUS Database Platform. Source: SCOPUS (2025) (www.scopus.com).

Carbone et al.^20^ state that green techniques, such as microwaves and ultrasounds, are more effective than traditional techniques in recovering secondary metabolites from medicinal plants as hops. Consequently, it is possible to suggest that the combination of green extraction techniques with green solvents can be adapted according to the desired target components. For instance, the bioactive compound recovered from plant materials using green methods can be further applied in food formulations or for improving food preservation.^21^ The incorporation of bioactive compounds in food processing is a major global trend for the food industries.^22^

According to Koh et al.,^15^ the use of natural deep eutectic solvents (NADES) as extraction solvents is an alternative with potential for application in various industrial segments. Additionally, green solvents can be combined with green extraction methods such as ohmic heating,^23^ ultrasound,^24^ microwaves,^25^ pressurized liquids,^11^ and supercritical technology^26^ in order to increase their extraction potential and expand their industrial application.

In their studies, Grisales-Mejía et al.^27^ extracted phenolic compounds from Hass avocado residues (epicarp and seed) using 10 NADES consisting of choline chloride combined with sugars and acids in combination and employing ultrasound (40 kHz for 30 min at 28 °C) and pressurized liquid extraction (103.4 bar for 20 min at 100 °C). The results suggest that this combination, especially under high-pressure conditions, contributes to the recovery of extracts with a high content of phenolic compounds.

He et al.^28^ evaluated the extraction of natural flavonoids (naringin and neohesperidin) from Jiang *Fructus aurantia*, achieving remarkable efficacy using a sustainable ultrasound-assisted extraction protocol (240 W of ultrasonic power for 72 min at 62 °C) combined with DES based on choline chloride and ethylene glycol. Also, Hong et al.^29^ address that ultrasound-assisted extraction (200 W of ultrasonic power for 80 min at 40 °C) with choline chloride-based natural deep eutectic solvent has proven effective for the extraction of solanesol from tobacco leaves. This developed method can be effectively applied to extract solanesol from residual tobacco leaves.

As discussed earlier, emerging green extraction methods provide an alternative tool for isolating bioactive molecules from agroindustrial waste. Several factors, including initial raw material, energy requirements, environmental impact, and quality of the resulting extract, influence the selection of the most suitable extraction technique and the adequate solvent.^21^ Notably, researchers and industries are increasingly focused on applying green extraction methods to address the growing demand for environmentally friendly and sustainable practices.^30^ In general, green solvents can be applied across various sectors of the processing industry, such as food, pharmaceutical, biorefinery, petrochemical, and others. There is also an exponential growth in the search for extraction with green solvents and their applications, which is supported by the great interest of the scientific community in emerging solvents (Figure 1).

## 3 DES and NADES Preparation and Their Role in Extracting Compounds from Renewable Materials and Waste

### 3.1 Deep Eutectic Solvents

DES represents a class of green solvents that can be utilized alongside green technology to prevent the generation of harmful effluents during the extraction of natural compounds.^31^ The synthesis of DES involves combining two compounds that engage in specific interactions, mainly hydrogen bonds, to form a eutectic mixture with a eutectic point lower than that of the individual components.^1^

Hydrophobic DES are primarily used for the extraction of inorganic and organic compounds from aqueous food samples, while hydrophilic DES are more suitable for the extraction of analytes from low-polarity food samples.^32^ Various external factors, including temperature, extraction time, ionic strength, and pH of the samples, can influence extraction efficiency.^33^

The continuous growth of research and applications related to DES has attracted considerable attention in recent years due to the new possibilities offered in various scientific disciplines, especially in the area of green analytical chemistry.^34^ The ″eutectic point″ refers to the intersection of the two liquid–liquid equilibrium curves of a binary system. At this point, three phases (one liquid and two solid) coexist, with both the composition and the melting point being fixed.^35^ DES has gained popularity and utility, with proven applications in extracting a wide variety of food components and in other industrial sectors.^36^ DES are broadly classified into five types: type I, quaternary ammonium salt + metal chloride; type II, quaternary ammonium salt + metal chloride hydrate; type III, quaternary ammonium salt + hydrogen bond donor; type IV, metal chloride hydrate + hydrogen bond donor; and type V, hydrogen bond acceptors (HBA) + hydrogen bond donors (HBD). The type V representing a unique category composed exclusively of nonionic molecular compounds that act as both HBD and HBA.^37^ DESs remain liquid at room temperature, are nonvolatile, exhibit excellent thermal stability, and can dissolve a wide range of organic and inorganic substances. These eco-friendly solvents have attracted significant attention and are expected to replace traditional organic solvents.^38^

An important factor to consider when selecting a solvent is its physicochemical affinity toward the target compound(s). DESs demonstrate the ability to extract bioactive compounds, with their effectiveness depending on the specific components of the eutectic mixture.^39^ The miscibility of the extracted components in DES is explained by their interactions, such as hydrogen bonds as well as van der Waals forces and ionic bonds.^40^

The ability of DES to form hydrogen bonds (through the transfer of protons and electrons) increases its dissolving power, which explains its effectiveness in extracting organic and inorganic compounds from food and biological samples.^33^ The synthesis of DES can be done assisted by various energy sources (Figure 2), such as conventional heating, microwave reactor, and ultrasound.^41^

**Figure 2:**
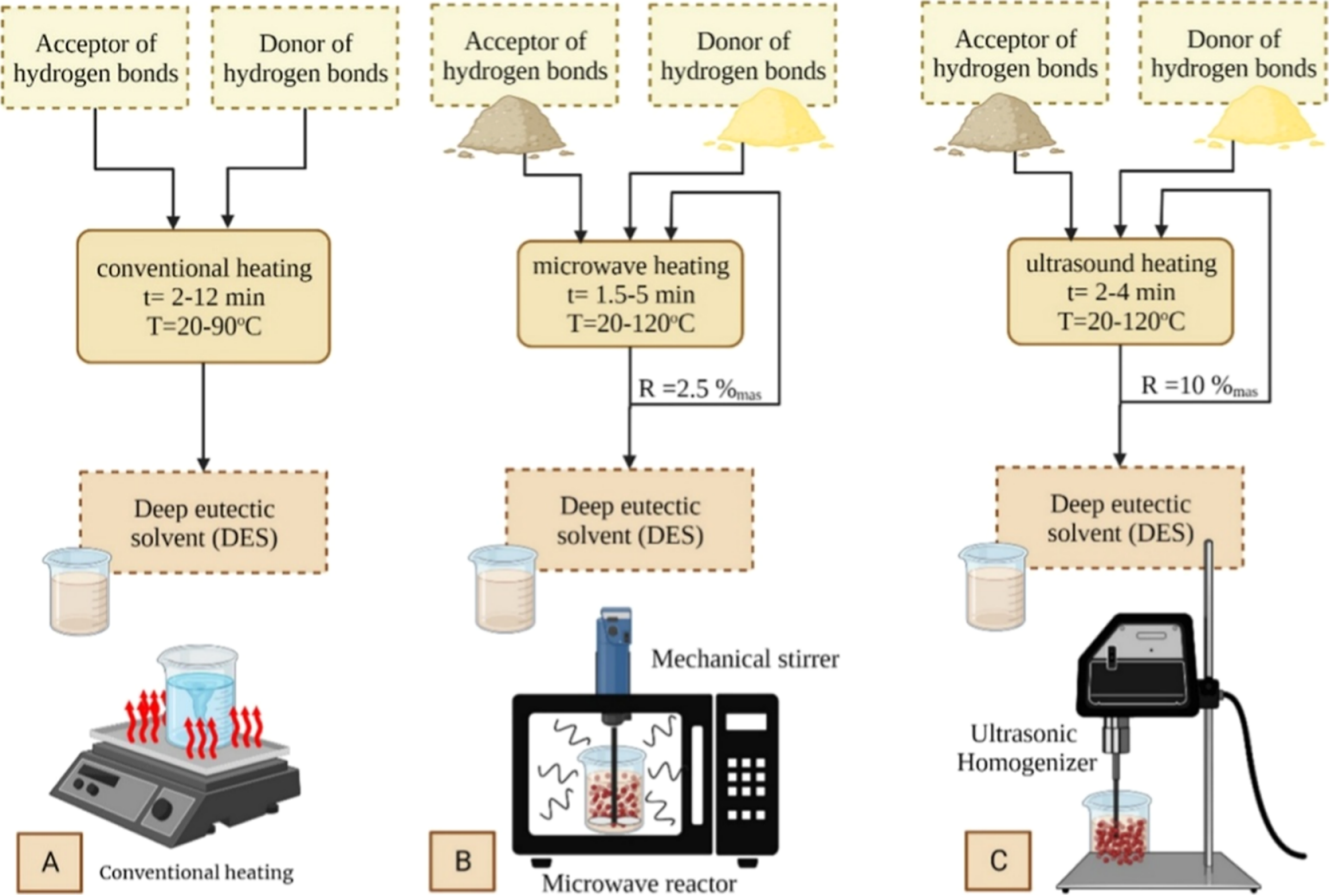
Schematic of the process of obtaining DES using conventional heating (A), microwave reactor (B), and ultrasound (C).^41^ Reprinted with permission from Długosz, O.; Banach, M. Green methods for obtaining deep eutectic solvents (DES). *J. Clean. Prod.* **2024**, 434, 139914. Copyright 2024 Elsevier.

For example, Sapyen et al.^42^ prepared DES by mixing choline chloride (HBA) and thymol (HBD) in a screw-capped vial in a molar ratio of 1:4. The prepared mixture was stirred in a hot water bath maintained at 70–80 °C until a clear liquid was obtained, indicating the successful preparation of DES. Similarly, Jiang et al.^43^ prepared binary DES by mixing thymol (HBA) with two HBD (menthol and octanoic acid) in a molar mass ratio of 1:1. This mixture was heated to 80 °C until a clear and transparent liquid was formed.

DES are applicable to electrochemistry, nanotechnology, and catalytic reactions and serve as potential solvents in the field of materials science.^44^ In several industrial sectors (chemical, electrochemical, biological, and biotechnology), DES has demonstrated remarkable potential.^45^ These solvents can address processing challenges at industrial scale, facilitating the recovery of diverse products from many renewable sources in order to produce dietary fibers and food packaging that contribute to the integration of a circular economy in the field and in industry.^46^

Researchers applied different ionic liquids (ILs) and DES to preserve biopharmaceutical delivery; interestingly, the results were similar regarding biopharmaceutical stabilization, having the potential to overcome current challenges in biopharmaceutical formulation.^47^ In the biorefinery sector, it is noted that the DES was used in the treatment of cellulose derived from plant fibers, achieving a deconstruction and separation efficiency of the main lignocellulosic components of 94% or more, selectively removing, depolymerizing, inhibiting polymerization and forming oligomeric lignin nanolignin particles.^48^ Finally, Sharma and Lee^45^ emphasize that the toxicity profile of DES depends on their nature, concentration, and interaction with living organisms. To reduce possible risks, it is necessary to carefully choose the components and concentrations.

### 3.2 Natural Deep Eutectic Solvents

Natural deep eutectic solvents (NADES) refer to a large class of natural biological metabolite complexes comprising the main monomeric components (sugars, organic acids, polyols, and choline derivatives).^49^ The growing application of NADES derived from renewable sources in analytical chemistry represents a progressive and sustainable development, being aligned with some of the 12 principles of Green Chemistry and the 17 Sustainable Development Goals of the United Nations.^3^

The composition of NADES is characterized by the system that constitutes a DES and the components are natural compounds.^50,51^ NADES offer notable advantages such as low toxicity, biodegradability, simple obtaining, and low cost. The melting point, density, viscosity, and polarity can be adjusted by changing the components and molar ratios.^52^

The significant increase in interest in NADES for the extraction of bioactive compounds from natural sources represents a progressive and sustainable development, mainly due to their potential to be derived from renewable sources.^3^ NADES can be prepared through several methods, including heating and stirring, vacuum evaporation, freeze-drying, microwave-assisted method, and ultrasound-assisted method.^15^ When NADES (liquid or solid) are mixed at elevated temperatures, hydrogen bonds form between the substrates, resulting in the transition to a liquid phase after a certain period.^53^

It is worth mentioning that the obtaining of NADES occurs similarly to DES, that is, using conventional heating, a microwave reactor, and ultrasound (Figure 2). For instance, Al Fuhaid et al.^54^ employed NADES in their studies, using dl-menthol, lactic acid, d-glucose, glycerol, octanoic acid, and betaine prepared in specific molar ratios using the heating method. Wu et al.^55^ prepared NADES via stirring and heating; in brief, a water bath with magnetic stirring heated to 60 °C was used until a homogeneous and transparent liquid was formed. Additionally, as noted by Pan et al.,^56^ NADES composed of choline chloride, lactic acid, citric acid, and malic acid were prepared by sonication for 15 min and heated at 80 °C until a clear homogeneous liquid was formed.

Extracts derived from agroindustrial waste using NADES can contribute to the principles of the circular bioeconomy since these extracts have the potential to be used in the development of new products.^27^ Socas-Rodríguez et al.^9^ tested NADES consisting of betaine as HBA with different HBD, and the combination of betaine:1,2-propylene glycol in a molar ratio of 1:4 provided the highest extraction efficiencies for the largest number of selected compounds (pesticides) under the optimized extraction conditions.

DES/NADES as green extraction solvents can be used for the recovery of anthocyanins and carotenoids (Figure 3) from raw materials.^10^ The use of emerging solvents such as DES/NADES for extraction processes has shown to be very promising, as a result of their high solvation capacity, in addition to having low or no toxicity.^2^ According to Figure 3, different extraction methods (microwave and ultrasound) can be employed with DES/NADES for the recovery of carotenoids or anthocyanins from waste due to the increasing demand for efficiently extracted natural pigments. In addition, it is mandatory to evaluate the safety/toxicity of the obtained extracts and their use in the food industry.

**Figure 3:**
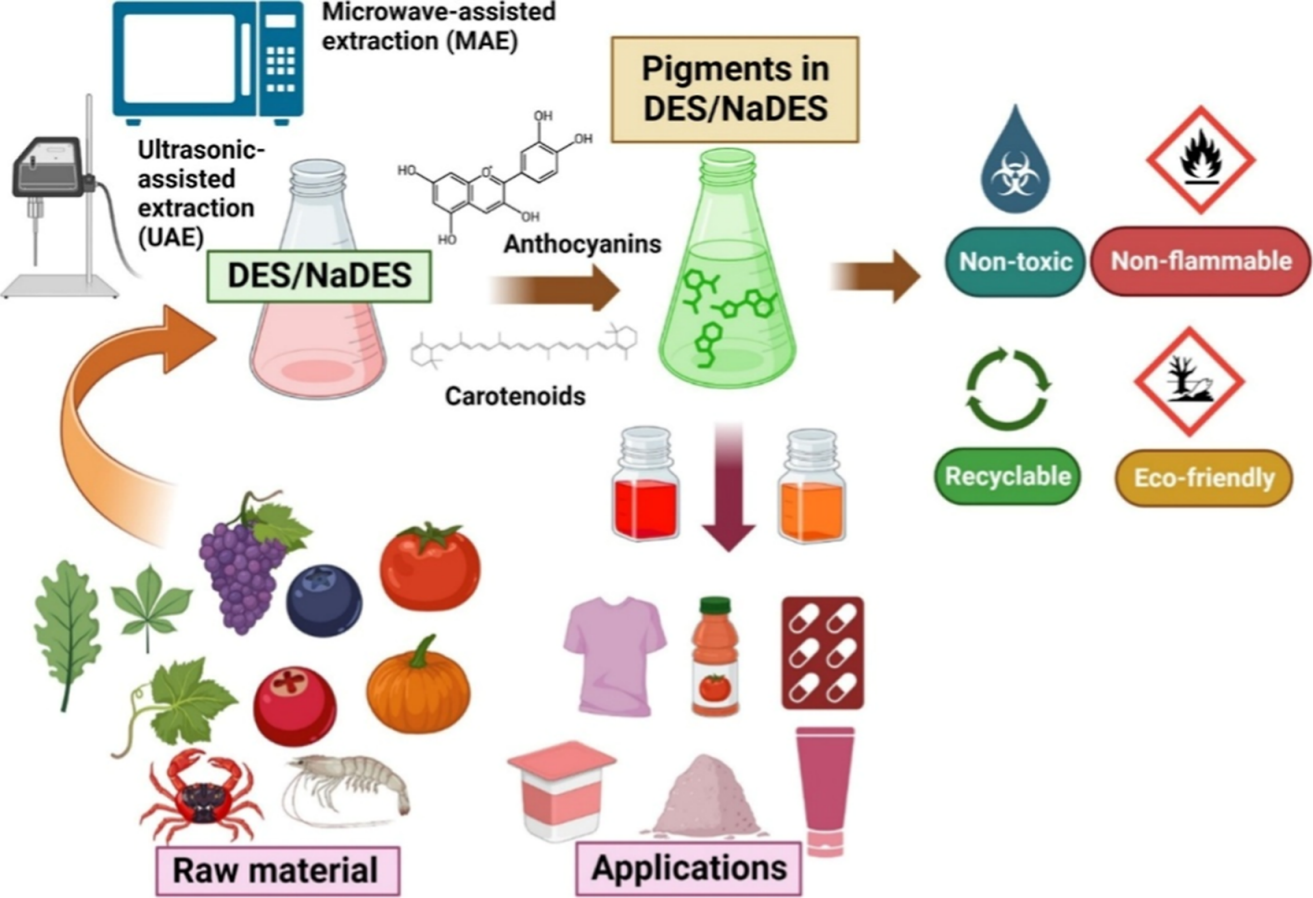
Schematic diagram of sources, extraction, and application of anthocyanins and carotenoids.^10^ Reprinted with permission from Airouyuwa, J.O.; Sivapragasam, N.; Redha, A.A.; Maqsood, S. Sustainable green extraction of anthocyanins and carotenoids using deep eutectic solvents (DES): A review of recent developments. *Food Chem.* **2024**, 448, 139061. Copyright 2024 Elsevier.

## 4 The Use of Monoterpenoids as DES/NADES for Green Extractions and Other Applications

Terpenes are a large class of simple hydrocarbons found in numerous plants, while terpenoids are terpene species containing different functional groups with oxygen.^57^ Among the terpene families, monoterpenoids consist mainly of two linked isoprene units.^58^ It is well known that certain terpenes and terpenoids have been utilized in the preparation of DES due to their hydrophobic nature, low environmental impact, and because these are not harmful, in addition to being proposed for the separation of organic compounds from aqueous streams.^59^

For example, menthol (waxy, crystalline, transparent, and solid substance at room temperature) is a cyclic monoterpene alcohol that can be either synthetically produced in a laboratory or extracted from plants belonging to the mint family (*Mentha piperita*) and generally used in foods as an additive.^60–63^ Additionally, 1,8-cineole (eucalyptol) is a natural compound derived from botanical sources such as eucalyptus and a predominant constituent of several essential oils from aromatic plants used in food, cosmetic, and pharmaceutical products.^64–67^

Thymol is a natural monoterpene phenolic compound found in several plants of the *Lamiaceae* family and is the main component of vegetable essential oils derived from these plants.^68,69^ Camphor, another monoterpene, has a bicyclic configuration and is obtained from natural origins.^70^ Additionally, other monoterpenoids such as geraniol, eugenol, carvone, linalool, citronellol, citronellal, citral geranial, carvacrol, and fenchone can also be employed and obtained from natural sources.^11,26,57^

Rozas et al.^11^ discuss that monoterpenoid-based DES exhibit moderately strong and highly localized hydrogen bonds, which are formed around the acceptor sites in monoterpenoids and develop in highly hydrophobic environments. The viscosity of NADES plays an important role in its selection for industrial use. Consequently, terpene-based mixtures have gained prominence due to their relatively low viscosity.^57^ These DES/NADES formed by monoterpenoids can be applied in extraction processes in the food industry for the extraction of bioactive compounds, natural pigments, and antioxidants from food residues or matrices. Moreover, they can also be applied in the pharmaceutical industry due to their potential for innovation in the health area. Furthermore, they can be utilized in the chemical and petrochemical industry for the treatment of effluents, biorefinery of compounds with high added value, metallurgy, and supercritical technology. Table 1 presents some recently selected examples of the applications of DES/NADES in different fields.

**Table 1:** Selected Application of Monoterpenoid-Based DES/NADES in Different Fields^a^

sample	extraction technique or method	DES/NADES composition (molar ratio)	target compounds/properties	analytical techniques and characterization	yield/recovery (%)	reference
Extraction of bioactive compounds						
dried Taxus chinensis needles	SL with heating, shaking methods and ultrasonic machine	menthol: propylene glycol (1:1); menthol: 1,2-butanediol (1:1); menthol: 1,3-butanediol (1:1); menthol: 1-propano (1:1); menthol: 1-butanol (1:1); menthol: 1-hexanol (1:1); menthol: 1-heptanol (1:1); menthol: 1-octanol (1:1); menthol: 1-nonanol (1:1) and menthol: decyl alcohol (1:1)	taxanes	HPLC	96.40 (10-DAB III); 95.33 (baccatine III); 98.97 (7-xyl-10-DAT); 96.14 (10-DAT); 99.28 (cephalomannine); 95.31 (7-*epi*-10-DAT) and 96.98 (Paclitaxel)	^23^
pretreated sweet sorghum silage press juice	reactive LL with tri–n–octylamine	thymol: menthol (2:7) to (7:2)	carboxylic acids	HPLC	-	^72^
real olive vegetation water	LL	menthol: camphor (1:1) and menthol: borneol (1:1)	phenolic compounds	HPLC	93.00 (at a solvent/feed ratio in volume of 3.00) and 88.73 (at a solvent/feed ratio by volume of 0.50)	^74^
orange peels	SL with UAE	menthol: eucalyptol (1:1) and menthol: camphor (1:1)	carotenoids	UV–Vis	-	^77^
Citrus sinensis peels	SL with UAE	menthol: eucalyptol (1:1)	carotenoids	UV–Vis	28.20	^24^
orange peel	SL with shaking method	menthol: camphor (1:1) and menthol: eucalyptol (1:1)	carotenoids and polyphenols	UV–Vis	-	^39^
orange peels	conventional, ultrasound and microwave independently and simultaneously	menthol: eucalyptol (1:1) and menthol: camphor (1:1)	carotenoids	UV–Vis	-	^25^
Ginkgo biloba leaves	naturally recyclable two-phase aqueous micellar system	menthol: formic acid (1:1); menthol: acetic acid (1:1); menthol: propionic acid (1:1); menthol: butyric acid (1:1); menthol: hexanoic acid (1:1); menthol: octanoic acid (1:1); menthol: dodecanoic acid (1:1); menthol: lactic acid (1:1); menthol: levulinic acid (1:1) and menthol: malonic acid (1:1)	phytochemicals	UHPLC–MS/MS	-	^75^
Application in the health and pharmaceutical areas						
orange (Citrus sinensis) peel	SL with UAE	menthol: eucalyptol (1:1)	biological properties	cytotoxicity, antiproliferative capacity, antioxidant capacity and antimicrobial potential	-	^77^
-	-	camphor: menthol (1:0.65), (1:0.97), (1:1.47) and (1:2.25)	solubility of cinnarizine	HPLC	-	^60^
-	-	menthol: myristic acid (8:1); menthol: lauric acid (4:1) and menthol: stearic acid (8:1)	antimicrobial potential evaluation	disk diffusion assay, assessment of minimal inhibitory concentrations, minimal bactericidal concentration, minimal fungicidal concentrations, biofilm formation and cytotoxicity assessment	-	^81^
chitosan films plasticized with thymol-based DES	-	octanoic acid: thymol (1:1); thymol: menthol (1:1) and octanoic acid: menthol: thymol (1:1:1)	mechanical and bioactive properties	thickness, morphology, structure, antioxidation assay, Antibacterial capacity and biocompatibility analysis	-	^43^
-	-	camphor: thymol (1:1) and camphor: menthol (1:2)	solubility of lidocaine	UV–Vis	-	^82^
isoniazid-free human blood	LL microextraction	thymol: 4-methoxybenzaldehyde (1:1); thymol: 2,4,5-trimethoxybenzaldehyde (1:1); thymol: 4-methylbenzaldehyde (1:1) and thymol: vanillin (1:1)	separation and determination of isoniazid	HPLC with UV–Vis	-	^83^
Chemical and petrochemical areas						
eggs and chicken tissue	liquid-phase microextraction combined with UV–Vis	thymol: menthol (1:1); thymol: hexanoic acid (1:2); thymol: heptanoic acid (1:2) and thymol: octanoic acid (1:2)	determination of selenium	atomic absorbance spectrometer AAS-7000	-	^84^
water	LL	menthol: lauric acid (1:1); menthol: decanoic acid (1:1) and menthol: octanoic acid (1:1)	separation of *tert*-butyl alcohol	chromatographic column	-	^87^
hydrochloric acid media	LL	menthol: lauric acid (1:1)	indium and thallium partition	PerkinElmer automatic gamma counter with an NaI detector	-	^85^
water	hollow fiber liquid phase microextraction	menthol: formic acid (1:2)	separation of triazines	HPLC	-	^71^
crude oil-polluted water	superhydrophobic melamine sponges impregnated	eucalyptol: menthol (1:5)	separation of oil	oil–water separation procedure based on standard test method IP 469/01	-	^86^
Biorefinery						
microalga spirulina (Arthrospira platensis)	SL with LL and triphasic solid–liquid–liquid	menthol: octanoic acid (5:1); menthol: lauric acid (4:3); menthol: 1; 2-octanediol (1:1); menthol: 1,3-propanediol (1:2) and menthol: thymol (1:1)	carotenoids, phycobiliproteins and free fatty acids	UV–Vis and liquid chromatography–mass spectroscopy	-	^89^
microalga Haematococcus pluvialis	SL	thymol: oleic acid (1:1) and geraniol: oleic acid (2:1)	carotenoid astaxanthin	HPLC with UV–Vis and HPLC–MS	-	^78^
alga Haematococcus lacustris	SL	menthol: acetic acid (1:1); menthol: lactic acid: water (3:3:1) and menthol: caprylic acid (1:1)	carotenoid astaxanthin	HPLC with UV–Vis	-	^54^
Metallurgy						
-	dispersive LL	choline chloride: thymol (1:4)	chromium speciation	inductively coupled plasma optical emission spectroscopy with an iCAP 6500 series ICP-OES spectrometer	-	^42^
magnetic nanoparticles deposited on a metal–organic framework	magnetic solid-phase with ultrasounds-assisted dispersive LL microextraction	menthol: stearic acid (4:1)	preconcentration of mercury(II)	electrothermal atomic absorption spectroscopy	-	^91^
Combined with carbon dioxide supercritical and thermophysical properties						
-	-	eucalyptol: thymol (1:1); eucalyptol: menthol (1:1); carvone: thymol (1:1) and carvone: menthol (1:1)	high-pressure carbon dioxide solubility	high-pressure gas sorption apparatus	-	^95^
solid cake from plant-based milk production	carbon dioxide supercritical	menthol:1,8-cineole (1:1) and camphor:1,8-cineole (1:2) used at concentrations of 7.5 and 15%	extracts rich in fatty acids, bioactive compounds, and a protein concentrate	phase behavior of the system supercritical, cytotoxicity, Soxhlet extraction, gas chromatography, and Kjeldahl method	-	^94^
-	-	carvone: linoleic acid (1:1) and eucalyptol: linoleic acid (1:1)	high-pressure gas capture of carbon dioxide and nitrogen, density, viscosity, conductivity, acidity and surface tension	high-pressure gas sorption setup, Anton Paar Density Meter DMA 1001, Anton Paar Rotational Viscometer ViscoQC 300, Mettler Toledo SevenCompact Duo S213 equipment and First Ten Angstroms Dynamic Contact Angle Analyzer FTA200	-	^96^
-	-	camphor: linalool (1:1); camphor: citronellol (1:1); camphor: geraniol (1:1); eucalyptol: linalool (1:1); eucalyptol: citronellol (1:1); eucalyptol: geraniol (1:1); carvone: eucalyptol (1:1); carvone: citronellol (1:1); carvone: geraniol (1:1); citronellal: eucalyptol (1:1); citronellal: citronellol (1:1); citronellal: geraniol (1:1); citral geranial: eucalyptol (1:1); citral geranial: citronellol (1:1) and citral geranial: geraniol (1:1)	physicochemical properties (density, shear viscosity, thermal conductivity, refractive index, and Reichardt’s polarity parameter)	Anton Paar DMA1001, electromagnetic VINCI Tech EV1000 viscometer, Decagon devices KD2 Thermal analyzer, Leica AR600 refractometer and Reichardt’s dye	-	^11^
-	-	carvacrol: capric acid (1:1); carvacrol: capric acid (1:1); thymol: capric acid (1:1); eugenol: capric acid (1:1); menthol: capric acid (1:1); fenchone: capric acid (1:1); camphor: capric acid (1:1); β-citronellol: capric acid (1:1); geraniol: capric acid (1:1) and linalool: capric acid (1:1)	viscosity, noncovalent interactions. Microstructure and characteristics	MDJ-600G densitometer, NDJ-8S digital rotational viscometer and visual molecular dynamics	-	^57^

a Note: High-performance liquid chromatography (HPLC), HPLC–mass spectroscopy (HPLC–MS), liquid–liquid (LL), solid–liquid (SL), ultrasound-assisted extraction, Ultraperformance liquid chromatography–tandem mass spectrometer (UHPLC–MS/MS), UV–Vis spectrophotometer (UV–Vis).

In summary, through a quantitative analysis of the use of monoterpenoids for the synthesis of DES/NADES in the 29 selected studies presented in Table 1, the menthol is the most frequently used monoterpene in the literature, being employed in approximately 82.76% (24) of the studies, present in all applications shown in the table. Eucalyptol (1,8-cineole) and thymol were used in approximately 34.48% (10) of the works and camphor in 31.03% (9). Of particular note are the menthol/eucalyptol DES/NADES used in 27.59% (8) of the studies and the menthol/camphor DES/NADES present in 20.69% (6) of the reports.

### 4.1 Extraction of Bioactive Compounds

Chen et al.^17^ reported the use of L-menthol, an economically viable natural compound, as a hydrogen bond donor (HBD) for the preparation of DES with 2-ethylhexylphosphonic acid mono-(2-ethylhexyl) ester at a ratio of 1:2 in extraction processes. Menthol-based NADES as HBA in a 1:2 ratio with formic acid have been proposed as an alternative to traditional organic solvents used in extractive processes.^71^ Fan et al.^23^ demonstrated that aqueous solutions of natural menthol-based DES are effective, nontoxic green solvents, with significant potential for extracting phytochemicals from plant materials. The yields of the seven main taxanes were 1.27–2.65 times higher than conventional organic solvents, with yields exceeding 95.31%, making it possible to recycle the menthol-based aqueous DES at least three times with perfect extraction efficiency for the target compounds.^23^ Airouyuwa et al.^10^ described a study screening 68 DES formulations for carotenoid extraction from orange peel, in which the menthol/camphor combination exhibited superior performance. Demmelmayer et al.^72^ investigated thymol/menthol-based DES in ratios from (2:7) to (7:2) as a green alternative for the extraction of carboxylic acids from a model solution and pretreated sweet sorghum silage juice, obtaining the highest extraction efficiencies and the greatest biocompatibility.

NADES based on hydrophobic compounds such as thymol, eucalyptol, and menthol have been used to extract more lipophilic compounds.^3^ Trenzado et al.^73^ investigated hydrophobic NADES formed by eucalyptol and decanoic acid (capric acid). Results indicate low viscosity and low-density fluid, in addition to confirming the formation of hydrogen bonds. NADES are capable of penetrating and disrupting the membranes of cellular structures due to their lipophilic nature. Fan et al.^57^ evaluated the relationship between viscosity and noncovalent interactions of terpenoid-capric acid NADES based on camphor, thymol, menthol, β-citronellol, eugenol, geraniol, linalool, fenchone, carvone, carvacrol, capric acid, and lauric acid. The study observed that ketone-based and alkenyl alcohol-based terpenoids acted as hydrogen bond acceptors (HBAs), whereas phenol-based terpenoids predominantly served as hydrogen bond donors (HBDs). The results emphasized that the low viscosity of the studied NADES was influenced by the number of hydrogen bonds, while high viscosities were attributed to increased van der Waals interactions.

In the studies conducted by Rozas et al.,^11^ eight monoterpenoids were utilized to prepare NADES, consisting of HBA (camphor, eucalyptol, carvone, citronellal, and geranial citral) and HBD (linalool, citronellol, and geraniol). All systems employed a molar ratio of 1:1. Hydrophobic fluids with low density and significantly low viscosity with physicochemical properties suitable for various technological fields were reported, resulting in important data for the development of natural, nontoxic, and cost-effective hydrophobic systems.

Rodríguez-Llorente et al.^74^ studied the liquid–liquid extraction of phenolic compounds from a type of olive oil mill effluent with terpenoids and terpene-based hydrophobic DES. The results demonstrated that geraniol, eucalyptol, and menthol/camphor eutectic solvent outperformed the conventional methyl isobutyl ketone and diisopropyl ether solvents. The menthol + camphor mixture obtained the best phenol extraction yields, with 93.00% at a solvent/feed ratio by a volume of 3.00 and 88.73% at a solvent/feed ratio by a volume of 0.50,^74^ yield values also close to those obtained by Fan et al.,^23^ demonstrating the high yield values in extractions involving DES/NADES containing monoterpenoids. Regarding the solvent reuse process, it was observed that these are stable after reextraction with an alkaline NaOH solution, where the results show the stability of the solvents, with the yields being maintained in the solvent reuse process.^74^ Wang et al.^75^ established a biphasic aqueous micellar DES system based on menthol in water for the extraction of phytochemicals (flavonoid glycosides, terpene lactones, and bioflavonoids) present in *Ginkgo biloba* leaves. The analyses performed indicated that the formation of menthol-based DES micelles in water provided large contact areas between the target compounds and the extraction solvent as well as that the aqueous DES system could be recovered. Srivastava et al.^76^ developed polar NADES composed of acetic acid and thymol (1:3) for the study of phytochemical extraction of *Aegle marmelos* leaves. The results obtained demonstrated that NADES polarity was between water and methanol and that the system provided lower viscosity and density compared to several known NADES.

Studies by Viñas-Ospino et al.^24,25,39,77^ compared orange peel extracts obtained with various green solvents, including vegetable oils, fatty acids, and DES. Results confirmed that the menthol/eucalyptol (Me/Eu) DES achieved carotenoid extraction yields comparable to those obtained with hexane. The Me/Eu mixture was formed by stirring and heating at 50 °C for 1 h until a clear liquid was obtained.^24^ The NADES Me/Eu (1:1) could also be obtained by stirring in a water bath at 60–80 °C until a clear liquid was formed.^77^ Me/Eu extract obtained from orange peels showed the highest carotenoid extraction yield (28,20%), in addition to presenting high stability during the storage period.^24^ Also, according to Viñas-Ospino et al.,^24^ the results presented identify this solvent as a viable and sustainable alternative to organic solvents through attractive extracts for use in enriching food products, resulting from improved carotenoid extraction combined with nontoxicity and greater stability of carotenoids.

Furthermore, these extracts provided with the highest antioxidant activity^39^ that could be due to the presence of carotenoids, to the antioxidant activity of menthol, and to possible synergistic effects between them.^78^ These results demonstrate that the obtained extract may have strong antioxidant activity than the desired compounds alone.^39^ The results found show that the extracts obtained can be included in final products without any additional separation or purification steps, thus being of great interest to the industry due to cost reduction.^77^

Hydrophobic DES are comparable to nonpolar solvents (acetonitrile or hexane); for instance, Me/Eu presented a low polarity, close to the value obtained for acetonitrile,^24^ explaining the efficiency for carotenoid́ extraction. Additionally, the viscosity of hydrophobic DES is lower than that of vegetable oils, facilitating the diffusion of the desired compounds.^79^ The viscosity of DES has an effect on the solubilization of the extracted compounds and, consequently, on their stabilization capacity.^77^

Viñas-Ospino et al.^25^ explain that carotenoids such as β-carotene and β-cryptoxanthin exhibit enhanced solubility in hydrophobic DES. Specifically, menthol as the HBA and eucalyptol and camphor as HBD exhibited the best activity coefficient values (with lower activity coefficient indicating higher carotenoid solubility in DES) for β-carotene and β-cryptoxanthin. In this sense, we can conclude that DES based on monoterpenoids can be used to recover valuable compounds with high added value from food and industrial waste, demonstrating their potential for several studies.

### 4.2 Health and Pharmaceutical Areas

Viñas-Ospino et al.^80^ evaluated the potential biological properties of orange peel extracts obtained with hydrophobic NADES, composed of menthol, dodecanoic acid, octanoic acid, l-proline, and eucalyptol. The evaluated properties included cytotoxicity, antioxidant capacity, antiproliferative capacity, and antimicrobial potential. The study demonstrated that the extracts made with octanoic acid/proline and menthol/eucalyptol exhibited the most favorable biological activities, highlighting their potential for applications in the pharmaceutical industry.

The results obtained by Oliveira et al.^81^ show that the use of menthol and saturated free fatty acids as NADES have great potential as antimicrobial agents for preventive and therapeutic applications in various clinical scenarios, including wound healing. Furthermore, NADES formed by camphor and menthol may be promising for developing an effective drug delivery system for cinnarizine.^60^ Jiang et al.^43^ prepared grape packaging films from chitosans, incorporating multifunctional bioactives with DES (thymol, menthol, and octanoic acid) at concentrations of 0.5–1.5%. The incorporation of these DES significantly increased the mechanical strength and hydrophobicity of the films, in addition to showing antibacterial activity, antioxidant activity, and biosafety.

In pharmaceutical sciences, Padilla et al.^82^ investigated the solubility of lidocaine in DES composed of camphor, thymol, and dl-menthol. The results indicated that lidocaine was significantly more soluble in these DES than in water, in addition to presenting stronger interactions with thymol molecules and weaker interactions with dl-menthol and camphor. In the field of personalized medicine, Meshcheva et al.^83^ studied a liquid microextraction approach based on DES composed of thymol and 4-methoxybenzaldehyde for the separation and determination of isoniazid in human plasma by HPLC with UV–vis detection. This novel procedure not only utilized natural reagents and minimized sample consumption but also enhanced sensitivity, facilitating the accurate determination of isoniazid at therapeutic levels in human plasma. These studies demonstrate the promising versatility of NADES made with menthol, eucalyptol, fatty acids, camphor, and thymol in the pharmaceutical and health fields, with applications ranging from improving the biological properties of plant extracts, use as antimicrobials, use in drug administration, and analytical methods in pharmaceutical sciences.

### 4.3 Chemical and Petrochemical Areas

According to Shishov et al.,^84^ DES based on terpenes (thymol/menthol 1:1) and fatty acids (thymol/hexanoic acid 1:2, thymol/heptanoic acid 1:2 and thymol/octanoic acid 1:2) were investigated as hydrophobic extraction solvents for the preconcentration of selenium as a colored chelate (piazselenol) formed with 3,3′-diaminobenzidine. This study represents the first DES-based microextraction procedure combined with UV–vis spectrophotometry for the determination of selenium in eggs and chicken tissue for application in food quality control laboratories. DES composed of thymol and menthol provided suitable conditions for the detection of the analyte through the developed method, which was successfully applied for the analysis of eggs and chicken tissue samples.

Some studies such as Tereshatov et al.^85^ investigated eutectic mixtures of dl-menthol (HBA) and lauric acid (HBD) in the proportion 1:1 for the extraction of indium and thallium from hydrochloric acid media. It was observed that at high acid concentrations, such a eutectic mixture demonstrated advantageous behavior in the extraction process. Similarly, Díaz-Álvarez et al.^71^ explored the use of NADES based on l-menthol: formic acid (1:2) as a supported liquid membrane for the hollow fiber liquid-phase microextraction of triazines in water. According to the analysis of the AGREEprep tool, the developed method may be a more environmentally friendly approach compared with other microextraction methods found in the literature.

Makoś-Chełstowska et al.^86^ described a new method for crude oil–water separation using superhydrophobic melamine sponges impregnated by deep eutectic solvents (MS-DES) composed of eucalyptol and menthol in a 1:5 molar ratio, showing a high real crude oil absorption capacity. Additionally, MS-DES materials demonstrated superhydrophobic properties, low density, high porosity, and excellent reusability. These findings suggest that MS-DES materials could serve as an effective alternative for cleaning crude-oil-contaminated water, highlighting a new application for DES/NADES.

Su et al.^87^ employed menthol as a hydrogen bond acceptor (HBA) and octanoic acid, decanoic acid, and lauric acid as hydrogen bond donors (HBDs) in a 1:1 ratio to investigate the extraction and separation of a tert-butanol alcohol (TBA) and water azeotropic system. The experimental results demonstrated that a menthol-based DES significantly enhances the extraction and separation of the petroleum additive TBA under standard experimental conditions. Similarly, Neni et al.^88^ studied the evaluation of the potential of DESs (based on choline chloride and menthol) as aggregation inhibitors for asphaltenes, identifying eight DESs as effective in dispersing asphaltenes, providing a promising basis for application in the petroleum industry. Thus, in the area of chemistry, it is possible to observe that the studies found in the literature address the use of NADES of thymol, menthol, and eucalyptol in the improvement of analytical techniques for food quality control and even in the extraction of metals and organic compounds to reduce the environmental impact of oil spills, offering solutions for the petrochemical industry.

### 4.4 Biorefinery

Regarding biorefinery processes, Hilali et al.^89^ studied the biorefinery scenarios for microalgae such as Spirulina (*Arthrospira platensis*) using NADES based on menthol: octanoic acid (5:1), menthol: lauric acid (4:3), menthol: 1,2-octanediol (1:1), menthol: 1,3-propanediol (1:2), and menthol: thymol (1:1). According to the results, the use of the three-phase approach allowed an increase in productivity for the extraction of chlorophylls and carotenoids, thus paving the way for a microalgae biorefinery based on the use of NADES. Similarly, Pitacco et al.^78^ developed hydrophobic DES based on thymol: oleic acid (1:1) and geraniol: oleic acid (2:1) to extract astaxanthin from the microalgae *Haematococcus pluvialis* without using any cell pretreatment. Their results indicated astaxanthin recovery rates of approximately 60% and 30% when applied to lyophilized biomass or directly to algal culture, respectively, over a 6 h period. Moreover, the antioxidant properties of these DES contributed to the improved astaxanthin stability.

Rodriguez-Llorente et al.^90^ described a simple flow-based procedure for the extraction and alkaline back-extraction of acetic acid from an aqueous feedstock, utilizing geraniol and eucalyptol as sustainable bioderived green solvents. These solvents were effectively employed in multiple extraction/back-extraction cycles. Similarly, Al Fuhaid et al.^54^ employed menthol-based hydrophobic NADES to extract astaxanthin from the algae *Haematococcus lacustris* at room temperature. Therefore, new sustainable means are offered for converting biomass or waste into valuable products with added value. NADES and DES, especially those composed of menthol, thymol, geraniol, and eucalyptol, emerge as promising and sustainable alternatives for biorefineries. Since these green solvents facilitate the extraction of bioactive compounds from microalgae and other biomasses, they present advantages such as better productivity, stability, and lower environmental impact compared to conventional solvents.

### 4.5 Metallurgy

From a metallurgical perspective, chromium is one of the most significant metals in the industry. Sapyen et al.^42^ developed a liquid–liquid dispersive extraction method as a nonchromatographic separation technique for chromium speciation. This method utilized DES composed of choline chloride and thymol (1:4). The extraction and removal efficiencies through this approach demonstrated the sustainability and reusability of DES. Additionally, Ragheb et al.^91^ developed a novel magnetic biosorbent for the preconcentration and extraction of Hg­(II) by employing a low-density hydrophobic deep eutectic solvent composed of l-menthol and salicylic acid (4:1) as the extracting and complexing agent. The results obtained indicated that the procedure was successfully applied to the determination of trace amounts of Hg­(II) in various samples. According to these studies, NADES based on monoterpenes such as menthol and thymol have shown interesting results in metallurgical applications; however, this potential can still be better explored, where future investigations could focus on evaluating the sustainability and economic viability of these innovative solvents in metallurgical processes.

### 4.6 Combined with Carbon Dioxide Supercritical and Thermophysical Properties

According to Pérez et al.,^92^ a relatively unexplored opportunity lies in the combination of DES/NADES with supercritical carbon dioxide. This technology has the capability of dissolved solvent and form a supercritical phase. However, studies exploring the phase behavior of DES/NADES in combination with carbon dioxide are limited,^92^ similar to the knowledge on the thermophysical properties of NADES under temperature and pressure conditions, both being necessary for its application in different technologies.^93^

Strieder et al.^94^ used terpenoid-based solvents such as menthol:1,8-cineole 1:1 and camphor:1,8-cineole 1:2 combined with supercritical carbon dioxide to obtain extracts rich in fatty acids, bioactive compounds, and a protein concentrate derived from almond and peanut biorefinery. These solvents at concentrations of 7.5% and 15% allowed an increase in the yield of bioactive compounds and lipids. It was also observed that this combination with supercritical carbon dioxide extraction made it possible to remove and reuse the solvent that was used. Other recent applications of DES and NADES using menthol were found in the literature. Pérez et al.^92^ determined that menthol and thymol form a DES mixture in a molar ratio of 1:1. Their study demonstrated that carbon dioxide exhibits substantial solubility in DES under moderate temperature and pressure conditions, highlighting its potential use for impregnating porous and polymeric substrates. In addition, the phase transition of the carbon dioxide and menthol:1,8-cineole 1:1 system presents a homogeneous liquid phase, which contributes to the greater solvation power and extraction yield with supercritical technology.^94^

Al-Bodour et al.^26^ studied NADES such as carvone, eucalyptol, thymol, and menthol with a molar ratio of 1:1 in relation to their carbon dioxide absorption capacity. The study demonstrated that carbon dioxide solubility increased with higher pressure and lower temperature, making them possible alternatives for carbon dioxide capture processes. In a follow-up study, Al-Bodour et al.^95^ evaluated the solubility and absorption kinetics of carbon dioxide in NADES composed of eucalyptol and lactic acid. The findings highlight the potential of these mixtures for the carbon dioxide capture process to reduce environmental impact. Alomari et al.^96^ highlights the potential of NADES combined with eucalyptol and linoleic acid (1:1) as effective means for carbon dioxide capture, proposing new studies at higher pressures and the evaluation of the scalability of these processes for future industrial applications.

Benito et al.^93^ examined the properties of NADES composed of menthol/thymol (1:1) under high-pressure conditions. The results showed the relationships between intermolecular forces and thermophysical properties, where the main characteristics (low-density and compressible fluid under high-pressure conditions) of NADES are maintained even under very high-pressure conditions. This underscores the critical role of hydrogen bonds in determining the properties of NADES. Additionally, Pérez et al.^92^ investigated the phase behavior in the vapor–liquid equilibrium (VLE) of the DES and carbon dioxide system, attributing it to the hydrogen bonding between menthol and thymol.

Sas et al.^97^ measured the densities of DES based on menthol and organic acids under high pressures and various temperatures. It was observed that DES formed with organic acids had higher density values compared to those formed by menthol. It was also observed that isothermal compressibility and isobaric thermal expansivity increase with increasing temperature and decrease with pressure for all of the studied systems.

Pérez et al.^92^ evaluated the VLE of different mixtures of carbon dioxide with l-menthol, thymol, and DES (menthol/thymol) (1:1) at 35–60 °C and 200 bar using a variable volume display cell. The results obtained demonstrated that the solubility of DES in carbon dioxide is lower than the solubilities of the individual components. However, carbon dioxide was found to be substantially soluble in DES under moderate temperature and pressure conditions, thus making it suitable for various supercritical fluid techniques. The results showed that the solubility of carbon dioxide in hydrophobic monoterpenoid-based NADES increased with increasing pressure and decreasing temperature. Harifi-Mood and Sarafrazi^98^ carried out experimental measurements and molecular dynamics simulations of carbon dioxide capture in hydrophobic NADES based on thymol and menthol. The best result was obtained for NADES composed of thymol and camphor (4:6). We conclude that the combination of DES/NADES formed by menthol, thymol, geraniol, 1,8-cineole, camphor, and carvone with supercritical carbon dioxide presents a range of promising opportunities in areas such as bioactive compound extraction, membrane impregnation, high-pressure phase equilibria, and carbon dioxide capture. Further studies on the phase behavior of these systems under high pressure are still needed, since understanding the thermophysical properties of these mixtures under different pressure, temperature, and composition conditions is essential to optimize and expand their technological applications.

However, there are potential limitations and disadvantages regarding the effective use of these DES/NADES in various fields since there are still few studies in the literature addressing topics such as the toxicity of these solvents as well as their economic disadvantages and environmental impact. Despite being known as nontoxic and natural, according to Halder and Cordeiro,^99^ the toxicity data available for DES are considerably lower than those available for IL, being scarce and complex, with only a few experimental works focused on the toxicological properties of DES. Studies on the environmental impact of these DES remain uncertain and require experiments with rigorous quantification.^100^ In addition, some characteristic physicochemical properties, such as polarity, viscosity, density, and surface tension of DES may limit their application.^101^ Even with the constant search for economically viable green solvents, it is noted that the use of green solvents remains complex and uneconomical on an industrial scale, with challenges involving scalability.^102^ Furthermore, these limitations demonstrate the potential for conducting new studies and experiments that contribute to the development and application of monoterpenoid-based DES/NADES in different areas.

## 5 Conclusion

This review concludes that emerging green extraction methods using solvents from renewable sources represent a promising alternative to traditional organic solvents. These green solvents can be effectively combined with advanced technologies, such as ohmic heating, ultrasound, microwave, and supercritical heating, to enhance extraction efficiency. DES/NADES are sustainable and environmentally friendly options produced by combining two compounds through hydrogen bonds, resulting in a eutectic mixture with a reduced melting point. Their preparation involves selection of the molar ratios, aided by energy sources such as conventional heating, ultrasound, and microwave reactors. Monoterpenoid-based DES/NADES are particularly effective as nontoxic green extraction solvents. These can be used as green solvents for various extractions and other applications, such as obtaining extracts from fruit and vegetable peels, extraction of natural compounds from raw materials or agroindustrial wastes, superhydrophobic melamine sponges, impregnating polymeric substrates, solubility in drugs, biorefinery with algae, and chromium speciation. Finally, the combination with supercritical carbon dioxide, in addition to the study of phase equilibrium and the knowledge of thermophysical properties under various temperature and pressure conditions, are little explored areas that present potential opportunities.

## Data Availability

No data were used for the research described in the article.
